# Dual stress, equivalent harm? hypothesizing on the type of interactions between waterlogging and high temperature

**DOI:** 10.3389/fpls.2024.1472665

**Published:** 2025-01-27

**Authors:** Rocío A. Ploschuk, Roxana Savin, Gustavo A. Slafer

**Affiliations:** ^1^ Department of Agricultural and Forest Sciences and Engineering, University of Lleida – AGROTECNIO-CERCA Center, Lleida, Spain; ^2^ ICREA, Catalonian Institution for Research and Advanced Studies, Barcelona, Spain

**Keywords:** stress interactions, heat stress, flooding, yield, biomass, leaf physiology

## Abstract

Episodes of extreme weather, such as high temperatures and heavy rains causing waterlogging, have been becoming more frequent due to climate change, posing risks to crops and reducing growth and yield. While the impact of these stresses has been individually studied, there is a significant gap in understanding their combined effects within the same growing season. There were only 15 studies in the rigorous literature addressing the combined impact of high temperatures and waterlogging. None explicitly examined whether these combined effects were additive (penalties close to the sum of the individual penalties), synergistic (more severe penalties), or antagonistic (less severe penalties). We aimed to propose a sound hypothesis on the most likely type of interaction between these two stressors. Reviewing the scarce literature we found, against expectations, that antagonistic interactions were most common, followed by cases of additive effects, with synergistic interactions being rare. Notably, while the primary concern of virtually all studies was the impact on crop yield, most of them focused exclusively on leaf-level traits, whose responses did not correlate well with yield responses. This preliminary analysis provides solid roots for hypothesizing that waterlogging and high temperatures interact antagonistically; i.e., that plants might develop some resilience when exposed to one stress, potentially reducing the impact of the other. Should this hypothesis be accepted, considering not only physiological traits but also, and mainly, yield in major crops, there would be a less pessimistic view on the expected outcome of the increased frequency of crops being exposed to combined high temperature and waterlogging.

## Introduction

Due to climate change, the likelihood of croplands being exposed to different environmental constraints has increased over the past few decades (Zandalinas and Mittler, 2022; Palmgren and Shabala, 2024). Indeed, there has been a noticeable rise in the occurrence of extreme temperatures and intense rainfall (IPCC et al., 2023; Trnka et al., 2014).A recent report by a taskforce of academics, industry and policy experts presented evidence that the global food production will be at a growing risk due to extreme weather (the risk of a 1-in-100 years extreme weather event likely to increase to 1 in 30 by 2040; Bailey et al., 2015). Indeed, yield gaps tend to increase for major field crops globally (Gerber et al., 2024).

It has been documented for at least a couple of decades that more intense rainfall naturally leads to an increased frequency and severity of waterlogging events (Arnell and Liu, 2001), which might have been worsened by reduced soil drainage due to the exacerbated use of heavy machinery (Jackson, 2004). Waterlogging constrains crop performance by reducing soil oxygen levels, thereby lowering nutrient and water uptake by roots (Striker, 2012). As most crop plants are not adapted to anoxia, their growth is severely reduced or completely inhibited when grown in water-saturated soils (Colmer and Voesenek, 2009). Consequently, this abiotic stress causes significant economic losses, impacting over 12% of farmlands (Tian et al., 2021) and jeopardizing approximately 1,700 million hectares worldwide (Voesenek and Sasidharan, 2013), producing severe yield penalties in crops like wheat and barley if occurring immediately before flowering (de San Celedonio et al., 2014; Marti et al., 2015; Liu et al., 2020). For example, soil anoxia significantly reduced wheat yield in the French breadbasket region during the 2016 growing season, contributing to the largest yield decline in recent French history (Nóia Júnior et al., 2023a). Indeed, waterlogging has been ranked as the second leading cause of yield reductions in the US, and is reportedly responsible for annual global losses of around 74 billion USD (Kaur et al., 2020). The relevance of waterlogging in penalizing yields has increased so dramatically that there is a strong demand for more research on this issue (Nóia Júnior et al., 2023b).

As mentioned above, the higher frequency of storms responsible for increased waterlogging has been associated with rising temperatures (Myhre et al., 2019). There is no doubt that crops have been exposed to higher temperatures, with substantial yield losses expected as a result (Teixeira et al., 2013; Challinor et al., 2014), and the likelihood of crops encountering high-temperature scenarios has increased (Asseng et al., 2015; Gourdji et al., 2013; Kaushal et al., 2016; Zampieri et al., 2017). Indeed, the negative impact of high temperatures on yield of major crops has been recognized for many decades (Chinoy, 1947; Finney and Fryer, 1958), and recent literature provides quantitative predictions of yield loss per unit increase in mean temperature (e.g., Asseng et al., 2015). In addition to the rising mean temperatures, global warming will also increase the risk of heat waves (Lhotka et al., 2018; Pfleiderer et al., 2019). A heat wave is a weather phenomenon characterized by temperatures rising above expected values for several consecutive days (Kim et al., 2024). Heat waves can be particularly detrimental for crops, especially if they coincide with the critical period for yield determination (Carrera et al., 2024), when crop growth determines sink-strength (Reynolds et al., 2022). The likelihood of crops encountering high-temperature scenarios has increased (Asseng et al., 2015; Gourdji et al., 2013; Kaushal et al., 2016; Zampieri et al., 2017). Indeed, there has been a continuous upward trend in the frequency of extreme heat events. For instance, in Australia the number of days per year with extreme heat (those above the 99^th^ percentile) increased from less than 1 to more than 4 during the 20^th^ Century and was above 12 in the second decade of the 21^st^ Century (Palmgren and Shabala, 2024). In 2018, cereal production was reduced by 8% compared to the average of the previous 5 years due to the heat wave in Europe (Brás et al., 2021). An examination of yield data over a 50-year period (from 1964 to 2015) estimated that such extreme events led to average losses of 7.3% and 3.1% in cereal and non-cereal production, respectively, with the negative impact of this stress tripling over the last 50 years (Brás et al., 2021).

Most scientific studies have focused on evaluating the consequences of one of these abiotic stresses. However, as the frequency and intensity of both waterlogging and high temperatures -two of the four key abiotic stresses that threaten food production (Palmgren and Shabala, 2024)- increase simultaneously, it is becoming more common for crops to face these stresses together, either simultaneously or sequentially. When this happens, it is most likely expected that waterlogging and high temperatures will interact, amplifying their negative effects on crop yields. In other words, while each stress alone reduces yield, their combined effect could lead to a dramatic collapse in productivity, as these two stresses would synergistically penalize growth and yield. That expectation is grounded in the idea that the cumulative effects of multiple stressors are magnified by synergistic interactions (Brook et al., 2008). However, there is limited rigorous experimental evidence to unequivocally support such a compounding effect. Therefore, a crucial first step in understanding and predicting how crops respond to simultaneous exposure to waterlogging and high temperatures is to determine whether the resulting impacts are additive (the sum of individual effects), synergistic (greater than additive), or antagonistic (less than additive) (**Box 1**). Projects aiming to conduct such experiments would benefit from hypotheses informed by existing, albeit limited, scientific literature.

Box 1 **Additive, antagonistic and synergistic effects of combined stresses.**

**Figure B1:**
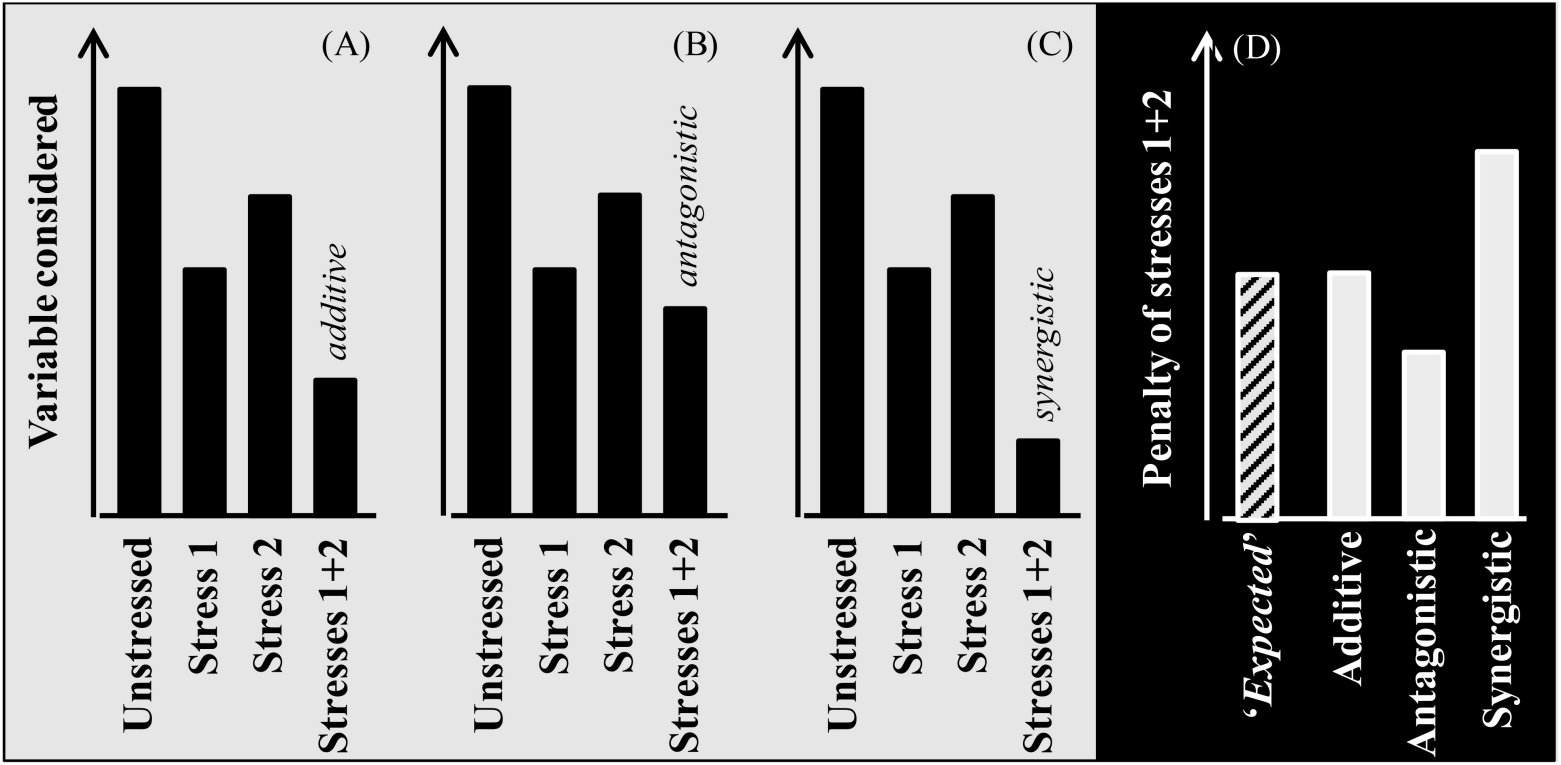
Scheme exemplifying different types of interactions between stresses when affecting the crop plants individually and combined. When the combined stress produces a penalty of a magnitude equivalent to the sum of the penalties imposed by each of them in isolation, their combined effect is additive **(A)**, suggesting that they act directly but would not interact. On the other hand, there are scenarios with interactions between stressors, being either antagonistic (when the mechanisms triggered by one of them do help plants to tolerate the other and therefore the combined effect has a magnitude smaller than the sum of the individual effects; **(B)** or synergistic (when the mechanisms triggered by one of them do increase the sensitivity of plants to the other and therefore the combined effect has a magnitude higher than the sum of the individual effects; **(C)**. Then, when comparing studies quantifying the effects of these stresses both in isolation and combined, it can be estimated the ‘expected’ additive effect (i.e. the sum of the penalties imposed by each stress in isolation) and comparing the actual and the ‘expected’ penalty produced by the combined stresses it can be determined whether the magnitude of the combined effect was actually additive (actual penalty similar to the expected) or if the stresses interacted as antagonistic or synergistic (when the actual penalty is less or more than the ‘expected’, respectively; **(D)**.

Due to climate change the likelihood of crops to be exposed to multiple stressors has, and will be, increased noticeably (Côté et al., 2016). Whenever plants are exposed to more than one stress, their combined effect may be additive, synergistic, or antagonistic [**Figure B1**; see also Zandalinas and Mittler (2022)]. The stresses are additive when their combined effect has a magnitude similar to the sum of the effects of each of the stresses when acting in isolation (**Figure B1A**). This implies that the two stresses do not interact: i.e. the mechanisms determining the observed response for each of the stresses are fully independent. The stresses are antagonistic if their combined penalty is less than that expected for additive stresses (**Figure B1B**). Antagonistic stresses trigger mechanisms of response that are similar for both stresses (or generally serving for adaptation or acclimation to different stresses). Finally, stress interaction is synergistic when the magnitude of the effect of the combined stresses is larger than the expected magnitude for an additive effect (**Figure B1C**). This implies that the mechanism(s) triggered in response to one stress, responsible for the penalty imposed by it, increase the damage imposed by the other stress. An example may be when drought and heat are combined: drought induce stomata closure (that avoid water loss, though penalizing growth) which increases tissue temperatures that, in turn, can more likely reach critical levels if the plants are also exposed to heat.

An additive effect would be more straightforwardly assumed, but chances are that there may be interactions between the responses to each of these stresses [as it has been shown for other stresses when combined (Barrett-Lennard, 2003; Gonzalez-Dugo et al., 2010; Slafer and Savin, 2018; Zhou et al., 2024)], so that the simplest additive assumption might not reflect the reality. According to the last interaction scenario, it was also found that stresses like high ozone and drought might present an antagonistic interaction, with lower negative impact of the stresses co-occurring than the sum of them acting separately (Iyer et al., 2013). On the other hand, the effect of two combined stresses such as drought and high temperature occurring simultaneously was found to be higher than the additive of each acting individually (Prasad et al., 2011; Savin and Nicolas, 1996). This is commensurate with the concept of co-limitation of limiting factors (Harpole et al., 2011; Sadras, 2004), with agronomic consequences where fertilizing with N may alleviate the penalty imposed by drought (Abid et al., 2016; Hu et al., 2008; Savin et al., 2022; Waraich et al., 2011). Heat and drought combined impair growth and yield more severely than expected from their individual effects (Sehgal et al., 2018). Moreover, it is known that plants subjected to a combination of stresses require a specific acclimation response, as in the case of drought and high temperature stresses (Mittler, 2006; Pandey et al., 2015; Rizhsky et al., 2004). Other combinations of abiotic stresses show conflicting results with some evidence supporting synergisms while other antagonisms. For instance, the effect of combined high temperature stress and nutrient deficiencies showed either synergistic (Sánchez-Bermúdez et al., 2022) or antagonistic effects (Slafer and Savin, 2018).

Whether the interaction between two stresses is antagonistic, synergistic, or additive seems to depend particularly on the specific stressors involved (e.g., see **Table 1** in Suzuki et al., 2014). Examples of potential additive, antagonistic, and synergistic effects of dual stresses are noted (see **Box 1**). However, the existing literature shows a strong bias toward synergistic interactions. For example, Côté et al. (2016) found that in a comprehensive review of studies on stressor interactions, synergistic effects were reported more than four times as often as antagonistic effects and more than twice as often as additive effects.

**Table 1:** List of the plant species, type of crop (either extensive field crops/pastures or intensive horticultural crops), number of genotypes and experimental conditions, waterlogging (WL) and high temperature (HT) treatments, indicating timing and durations, variables measured and references for data used in **Figures 1**–**3** and **Table 2**.

Species	Type of crop	Genotypes and conditions	Treatments (order of stresses)	Variables measured	Reference
Cotton	Extensive	1 genotype. Field plots (4x4m). 3 years	HT and WL started simultaneously from flowering. WL for 3 or 6 days, HT until boll maturity. Six treatments: control, WL_3d_, WL_6d_, HT, WL_3d_+HT, WL_6d_+HT. (Intermittent stresses: first simultaneous stresses for 3 or 6d, then only HT until maturity).	Pre-dawn leaf water potential. Enzyme activity (sucrose synthase, sucrose phosphate synthase). % cellulose and sucrose in fibers. Gene expression	Chen et al. (2017a)
				Photosynthesis. Fiber length. Sucrose, malate, and potassium content in fibers. Enzyme activity (sucrose synthase, PEPC and vacuole invertase). Gene expression	Chen et al. (2017b)
				Photosynthesis. Boll and seed biomass. Sucrose and starch content in fibers. Enzyme activity (Rubisco, sucrose synthase, sucrose phosphate synthase). Gene expression	Wang et al., (2017)
				Root and shoot dry weight. Nitrogen, chlorophyll a and b leaf content. Amino acid and soluble protein leaf content. Enzyme activity (nitrate reductase, glutamine and glutamate synthases, protease, and peptidase). Gene expression	Wang et al. (2018)
				Boll load (yield). Leaf area and number per plant. Chlorophyll a and b leaf content. Malondialdehyde (MDA), singlet oxygen (^1^O_2_) and hydrogen peroxide (H_2_O_2_) leaf contents. Enzyme activity [superoxide dismutase (SOD), catalase (CAT), peroxide (POX), glutathione reductase, ascorbate peroxidase].	Wang et al. (2019)
				Seed biomass. Percentages of oil, protein, and carbohydrates in seeds. Sucrose, starch, glucose, and fructose seed content. Enzymes (PEPC, G6PDH). Percentages of saturated and unsaturated fatty acids.	Xu et al. (2021)
Cotton	Extensive	1 genotype. Pots (20 cm height, 3 L capacity). 1 year	WL for 3 days that started when seedlings had their first leaf. HT for 30 days, imposed after 2 days of the end of WL. Four treatments: control, WL, HT and WL+HT. (Sequential stresses, first WL, then HT).	Root and shoot dry weight. Leaf area and number per plant. Chlorophyll a and b leaf content. Malondialdehyde (MDA), singlet oxygen (^1^O_2_) and hydrogen peroxide (H_2_O_2_) leaf contents. Enzyme activity [superoxide dismutase (SOD), catalase (CAT), peroxide (POX), glutathione reductase, ascorbate peroxidase].	Wang et al. (2024)
Maize	Extensive	1 genotype. Plastic tubes (150cm height) in field. 2 years	HT and WL simultaneously for 6 days applied at V3, V6 or VT (three-leaf, six-leaf, and panicle emergence stages, respectively). All in all, ten treatments were imposed: control, WL_V3_, WL_V6_, WL_VT_, HT_V3_, HT_V6_, HT_VT_, WL+HT_V3_, WL+HT_V6_, WL+HT_VT_. (Simultaneous stresses)	Yield, kernel number and weight. Shoot dry weight. Plant and ear height. Stem diameter. Number and area of vascular bundles (vessels). Lignin accumulation. Lignin-synthesis enzymes.	Shao et al. (2023)
				Photosynthesis, leaf greenness (SPAD), leaf area index (LAI). MDA, ascorbic acid leaf contents. Enzyme activity (Rubisco, PEP carboxilase, SOD, CAT, SOD).	Shao et al. (2024)
Ginger	Intensive	1 genotype. Pots (30cm diam, 25cm height) in growth chambers. 1 year.	HT and WL simultaneously for 60 hours applied with Four-five branches (and plant height of 60cm). Four treatments: control, WL, HT and WL+HT. (Simultaneous stresses)	Leaf water and chlorophyll contents. MDA, ^1^O_2_ and H_2_O_2_ leaf contents. Sucrose and reducing sugar leaf contents. Leaf fluorescence (Fv/Fm).	Liu et al. (2023)
Hot pepper	Intensive	1 genotype. Plots in glasshouse. 1 year.	HT applied from 14 DAS for 173 days. WL applied from 54 DAS for 72 hours. Four treatments: control, WL, HT and WL+HT. (Intermittent stresses: first HT, then HT and WL simultaneously, lastly HT only)	Yield, fruit number and weight. Plant height. Leaf area per plant. Leaf greenness (SPAD). Plant dry weight. Photosynthesis. Stomatal conductance. Water use efficiency.	Lee et al. (2017)
Cauliflower	Intensive	3 genotypes. Pots in growth chambers. 1 year	HT and WL simultaneously for 6-96 hours applied at 30 days DAS. Four treatments: control, WL, HT and WL+HT. (Simultaneous stresses)	Chlorophyll content and fluorescence, leaf water potential. Electrolyte leakage. Protein expression.	Lin et al. (2015a)
Broccoli	Intensive	2 genotypes. Pots (12.7 cm diam) in growth chambers. 1 year.	HT and WL simultaneously for 72/144 hours applied at 40 days after sowing (DAS). Four treatments: control, WL, HT and WL+HT. (Simultaneous stresses)	Leaf greenness (SPAD) and stomatal conductance. H_2_O_2_ leaf content. Protein and gene expression.	Lin et al. (2015b)
Tomato	Intensive	2 genotypes. Pots in growth chambers. 1 year	HT and WL simultaneously for 96 hours applied at 60 days DAS. Four treatments: control, WL, HT and WL+HT. (Simultaneous stresses)	Chlorophyll content and fluorescence.	Lin et al. (2016)
Kentucky Bluegrass (*P. pratensis*)	Extensive	2 genotypes. Pots (15cm diam, 14.5cm height) in glasshouse. 1 year.	HT and WL simultaneously for 5 days applied at 75 DAS. Four treatments: control, WL, HT and WL+HT. (Simultaneous stresses)	Shoot dry weight. Leaf elongation rate. Root water soluble carbohydrate and protein concentrations. Enzymes (alcohol dehydrogenase and lactate dehydrogenase). Paper reported a single value for the 2 genotypes.	Wang and Huang (2004)

There appears to be a scarcity of studies that analyze the responses of crop plants to waterlogging and high temperatures both individually and in combination. This may be due to (i) the historically low likelihood of these stresses occurring simultaneously, and (ii) the challenges of applying high temperature treatments under field conditions and waterlogging in experimental approach. To the best of our knowledge, no attempt has been made to synthesize the limited information scattered across the literature to identify discernible patterns that could support the development of a robust hypothesis about the interactions (or lack thereof) between waterlogging and high temperatures. Therefore, our objective was to propose a well-founded hypothesis on the most likely type of interaction between these two specific stressors. To achieve this, we reviewed the literature to identify studies that addressed the combination of waterlogging and high temperatures. By examining these studies together, we aimed to uncover any consistent patterns that could provide an empirical basis for hypothesizing whether the effects of these stresses are more likely to be additive, synergistic, or antagonistic.

## Searching the literature and building a database

A survey was conducted using the Web of Science database on February 28^th^, 2024. We searched for papers that (i) fell under the categories of ‘plant sciences’, ‘agronomy’, and ‘horticulture’, (ii) reported the effects of waterlogging and high temperatures separately and together, (iii) were published in journals ranked within the top three quartiles of the Journal Citation Report, (iv) had titles containing either ‘waterlogging AND high temperature’, ‘waterlogging AND elevated temperature’, ‘waterlogging AND heat’, ‘flooding AND high temperature’, ‘flooding AND elevated temperature’, or ‘flooding AND heat’.

The search results confirmed our expectation that there were very few studies published, particularly in mainstream literature, considering the combination of waterlogging and high temperatures on agricultural plant species, despite extensive individual studies on each stressor, especially high temperatures (see **Supplementary Figure S1** in the **Supplementary Material**). The search yielded a total of 28 papers, of which only 15 articles met the inclusion criteria for this study; the remaining 13 did not report results on yield, biomass, or specific physiological parameters (such as leaf greenness, chlorophyll fluorescence, leaf area), sugar metabolism (soluble sugar content, starch content, enzymes related), or oxidative metabolism (reactive oxygen species, detoxification enzymes). Among the selected papers, ten focused on plants of extensive agricultural crops, with relatively lower water inputs (crops: cotton and maize, and a pasture: Poa pratensis, ‘Kentucky bluegrass’), and five on plants of horticultural crops commonly produced under intensive agricultural systems, with higher water inputs (ginger, broccoli, hot pepper, tomato, and cauliflower), and none reported data for trees (see **Table 1**). Well after the search was finished (in June 2024) a new article was published reporting the effect of waterlogging and high temperature in cotton (Wang et al., 2024) and we included it in our database (see **Table 1**). Additionally, we found that most studies focused on the combined impact of waterlogging and high temperatures as simultaneous stresses, while only two studies reported findings on intermittent stresses and only one addressed sequential stresses (see **Table 1**).

Consistently with our initial presumption stated in the Introduction, our search revealed not only a scarcity of studies assessing the impact of waterlogging and high temperature treatments both individually and in combination, but also a limited number of studies were carried out until crop maturity. This limited scope hindered the evaluation of effects on both specific physiological processes and reproductive output, crucial for determining yield in grain crops. Indeed, most of the research conducted on these stresses, whether applied independently or in combination: (i) focused on biochemical-physiological processes (e.g., activity of enzymes with antioxidant functions or related to sugar metabolism; chlorophyll content, stomatal conductance, and leaf photosynthetic rate), and/or (ii) considered only the leaf level of organization (e.g. chlorophyll content, stomatal conductance, and leaf photosynthetic rate) (**Table 1**). Only two of the publications included data on yield, which is essential for understanding the agronomic implications of these stresses and underscores their relevance in field crop studies.

For analyzing the reported data comprehensively across different papers, the variables were categorized into three main sets of attributes: (i) yield, which is the most critical trait for assessing the agronomic relevance of the stresses and a common focus in crop physiological studies, (ii) shoot biomass (either whole plant or shoot only), as stresses often impact yield through effects on growth, and (iii) traits at the organ or sub-organ level of organization, which are commonly assessed in plant physiological studies. This latter set of attributes includes: (a) leaf physiology (such as photosynthesis, stomatal conductance, Fv/Fm, leaf greenness, chlorophyll content, Rubisco activity, and leaf area), (b) carbohydrates and enzymes related to carbohydrate metabolism (such as sucrose and starch leaf content, sucrose synthase, and sucrose phosphate synthase), and (c) derivatives of oxidative stress [such as malondialdehyde (MDA) content, reactive oxygen species (ROS) content including singlet oxygen and hydrogen peroxide, and activities of detoxification enzymes like catalase, peroxidase, and superoxide dismutase].

We excluded from the analysis any cases in which the measured variables showed a positive impact of high temperatures or waterlogging, as in those exceptional cases the treatments could not be considered stresses (Boyer, 1982). Therefore, studies were considered valid for inclusion in this work when the measured variables demonstrated a negative impact indicative of stress. This included reductions in yield, biomass, leaf physiology, and enzymes related to carbohydrate metabolism when plants were subjected to waterlogging, high temperatures, or both stresses combined. Additionally, increases in levels of reactive oxygen species (ROS), malondialdehyde (MDA), and enzymes related to detoxification were considered indicative of stress under waterlogging and/or high temperatures, as these compounds typically increase in response to plant stress (Inzé and Van Montagu, 1995; Hossain et al., 2015; Komatsu et al., 2011).

To analyze the data and infer the type of interaction between waterlogging and high temperatures for each variable considered in each experiment, we:

- assessed the penalty imposed by each stress individually (the relative difference between either waterlogging (WL) or high temperatures (HT) and the unstressed control for each variable within each experiment),
- calculated the ‘expected reduction’ if the combined effects were purely additive (simply the sum of the reductions caused by each stress when applied individually; see **Box 1**),
- evaluated the actual penalty imposed by the combined stresses (the relative difference between the combined treatment and the unstressed control for each variable), and finally
- compared the actual and expected relative changes for each variable in each experiment.

We categorized the combined effect as (iv.a) additive if the actual change fell within ±15% of the ‘expected change’, (iv.b) antagonistic if the actual change was less than 85% of the expected additive change, or (iv.c) synergistic if the actual change was more than 15% higher than the expected additive effect. The threshold of ±15% of the expected additive effect was used to categorize interactions as antagonistic (<15% below the expected change) or synergistic (>15% above the expected change), which was deemed a reasonable approximation. Choosing a lower threshold would have strengthened the conclusions.

## Crop yield

Unfortunately, despite an extensive literature search, very few studies were found that reported on both the isolated and combined effects of waterlogging and high temperatures on yield. Only two papers documented effects on yield, which is critical when assessing the agronomic impact of stress. These studies included a 3-year investigation with cotton (Wang et al., 2019) and a 2-year study with maize (Shao et al., 2023).

In the cotton study, stresses were applied simultaneously at flowering, followed by a period of HT only. After treatments, yield consistently decreased under waterlogging conditions (with a non-significant trend after 3 days of waterlogging and significantly after 6 days) and also decreased under high temperature conditions (significantly in two out of three growing seasons; see **Table 2**, left). When both stressors were applied simultaneously, the yield either did not decrease significantly, or showed only a slight reduction, compared to when each stressor was applied individually (see **Table 2**, left).

**Table 2:** Yield of cotton (left) and maize (right) grown under either unstressed conditions (Control) or stressed by exposing the plants to waterlogging (WL), high temperatures (HT) and their combinations (WL+HT)].

Cotton yield (g m^-2^)				Maize yield (g plant^-1^)		
Treatment	Year			Treatment	Year	
	1	2	3		1	2
Control	187.1a	175.2a	180.3a	Control	305.6a	265.3a
HT	167.2c	163.2b	169.2ab	WL_V3_	198.6e	199.1d
WL_3d_	180.6ab	170.0a	176.1a	HT_V3_	250.3cd	233.7b
HT+WL_3d_	177.6b	171.2a	175.3a	WL+HT_V3_	149.2f	184.4e
WL_6d_	155.3d	150.6c	153.2c	WL_V6_	208.8e	217.1c
HT+WL_6d_	150.3d	140.3d	143.0d	HT_V6_	283.8b	244.4b
				WL+HT_V6_	202.2f	197.5d
				WL_VT_	267.1c	242.4b
				HT_VT_	123.2f	91.2f
				WL+HT_VT_	107.6g	56.6g

In cotton, WL and HT were imposed together at flowering; WL lasted for 3 d (WL_3d_) or 6 d (WL_6d_), while HT remained until maturity.In maize, both WL and HT were imposed for 6 d simultaneously at three different stages of development (3-leaf, 6-leaf, and tasseling indicated as V3, V6 and VT, respectively). Different letters indicate significant differences (P<0.05) among treatments within each experimental year. Adapted from Wang et al. (2019) and Shao et al. (2023).

In the maize study (where waterlogging and heat stress were simultaneously imposed for 6 days at stages V3, V6, or VT; Ritchie and Hanway, 1982), yield was consistently significantly lower than the unstressed control when either of the two individual stresses was applied independently, across all timings and growing seasons (see **Table 2**, right). Simultaneous imposition of both stresses consistently resulted in a significant reduction in yield not only compared to the unstressed control but also compared to conditions where only one of the stresses was applied (see **Table 2**, right).

**Figure 1:**
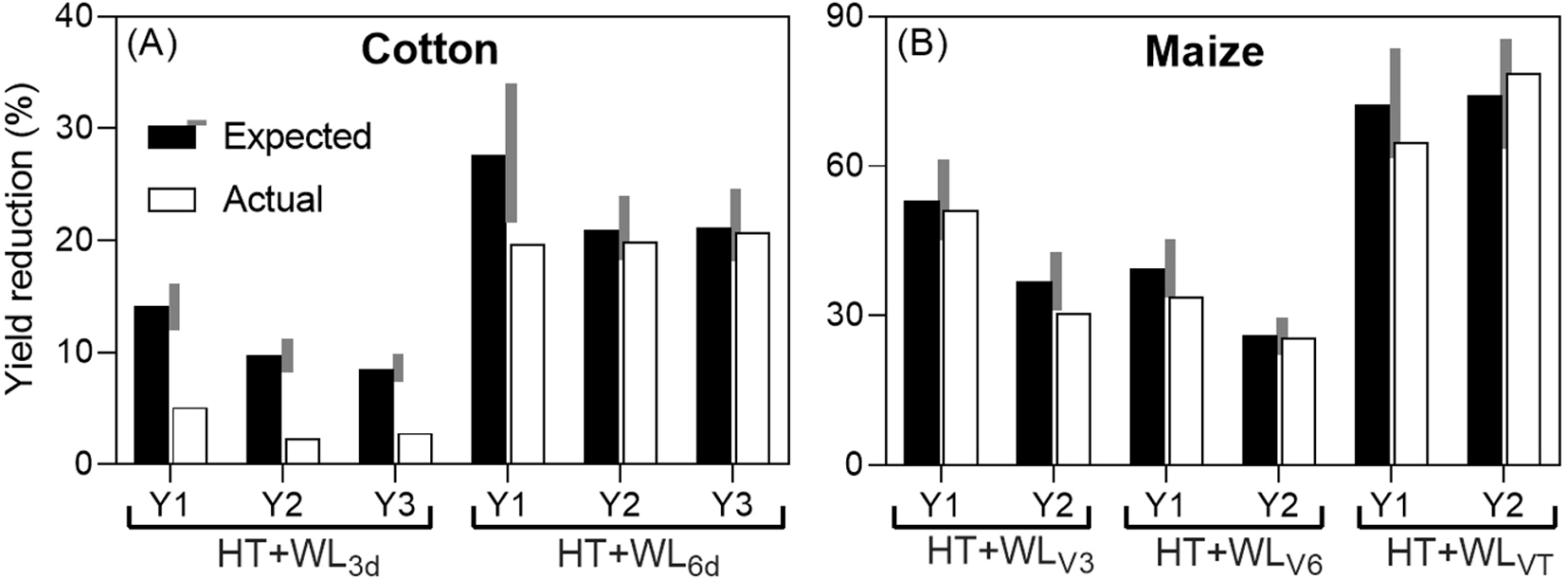
Expected additive (sum of the penalties due waterlogging and high temperatures when they were the sole treatments; see **Box 1**) and actual yield reductions due to the combined stresses (% of their respective controls; actual yields in **Table 2**) in cotton, subjected to intermittent stresses: first simultaneous stresses (WL+HT), followed by HT only **(A)** and maize, subjected to simultaneous stresses (WL+HT) **(B)**. The grey area on the side of the expected yield reduction represents a ±15% of its value and the effect was considered additive if the actual reduction was within this range, and antagonistic or synergistic if lower or higher than the grey area, respectively. The study with cotton had 3 experimental years (Y1, Y2 and Y3), with two waterlogging durations (being the combined treatments HT+WL_3d_ and HT+WL_6d_). In maize, there were 2 experimental years (Y1 and Y2), with the treatments starting in V3 (three-leaf stage, HT+WL_V3_), V6 (six-leaf stage, HT+WL_V6_) or VT (panicle stage or tasseling, HT+WL_VT_). Data were taken from Wang et al. (2019) and Shao et al. (2023).

When comparing for the two studies that reported effects on yield the actual reduction caused by the combined effect of waterlogging and high temperatures with the expected reduction (assuming additive effects of both stresses), it was found that in 5 out of the 12 cases analyzed, the effects were antagonistic (see **Figure 1**). Antagonistic effects were observed consistently when waterlogging lasted for only 3 days in cotton and in the first year when it lasted for 6 days, as well as in the second year of the maize study at stage V3. In all other cases, the effects were additive (see **Figure 1**), although there was a tendency towards antagonism: in 6 out of 7 cases classified as additive, the actual reduction was less than expected, but not by more than 15%. Notably, there were no instances of synergistic interaction (see **Figure 1**).

The absence of synergistic effects of waterlogging and high temperatures on yield, observed consistently across different stress durations (in cotton) or timings (in maize) across multiple seasons, contrasts with common expectations. Typically, one would anticipate a synergistic interaction between stresses, where the combined effect of stresses leads to a more severe impact on plants than the sum of the individual stresses. This synergistic effect is often observed with combinations such as heat-drought (Prasad et al., 2011; Suzuki et al., 2014; Zandalinas and Mittler, 2022) and heat-salinity (Suzuki et al., 2016). The rationale behind this expectation is that a stress would exacerbate yield penalties in crops already affected by another stress, due to increased vulnerability of weakened plants (Prasad et al., 2008). The absence of synergistic interactions in the limited available data also suggests a less pessimistic outlook regarding potential yield losses due to climate change. While the impact will undoubtedly be negative, it may not be as catastrophic as anticipated if synergistic interactions were prevalent.

The differences observed in combined stress effects on cotton and maize yields highlight that the nature of these interactions can vary depending on species, stress duration, timing, and sequence (Zandalinas and Mittler, 2022). This variability suggests that genotype interactions with stresses may also exist, opening up possibilities for breeding resilience not only to waterlogging or high temperatures individually but also to their combined occurrence.

## Biomass

During the survey, only 15 cases of shoot biomass assessment were identified across four papers. Thirteen of these cases were from studies on cotton and maize, where yield was also measured in twelve of those cases (see **Figure 1**), while the remaining two cases came from studies on hot pepper (Lee et al., 2017) and Kentucky bluegrass (Wang and Huang, 2004), each reporting only one case (see **Table 1**). Additionally, 7 of these cases corresponded to intermittent stresses, in the case of cotton, it was a combination of simultaneous stresses (WL+HT) followed by a period of HT only; while in hot pepper, first HT was applied, then HT and WL simultaneously, lastly HT only. Then, 7 of the rest of the cases corresponded to simultaneous stresses (maize and Kentucky blue grass) and 1 to sequential stresses (cotton; Wang et al., 2024).

**Figure 2:**
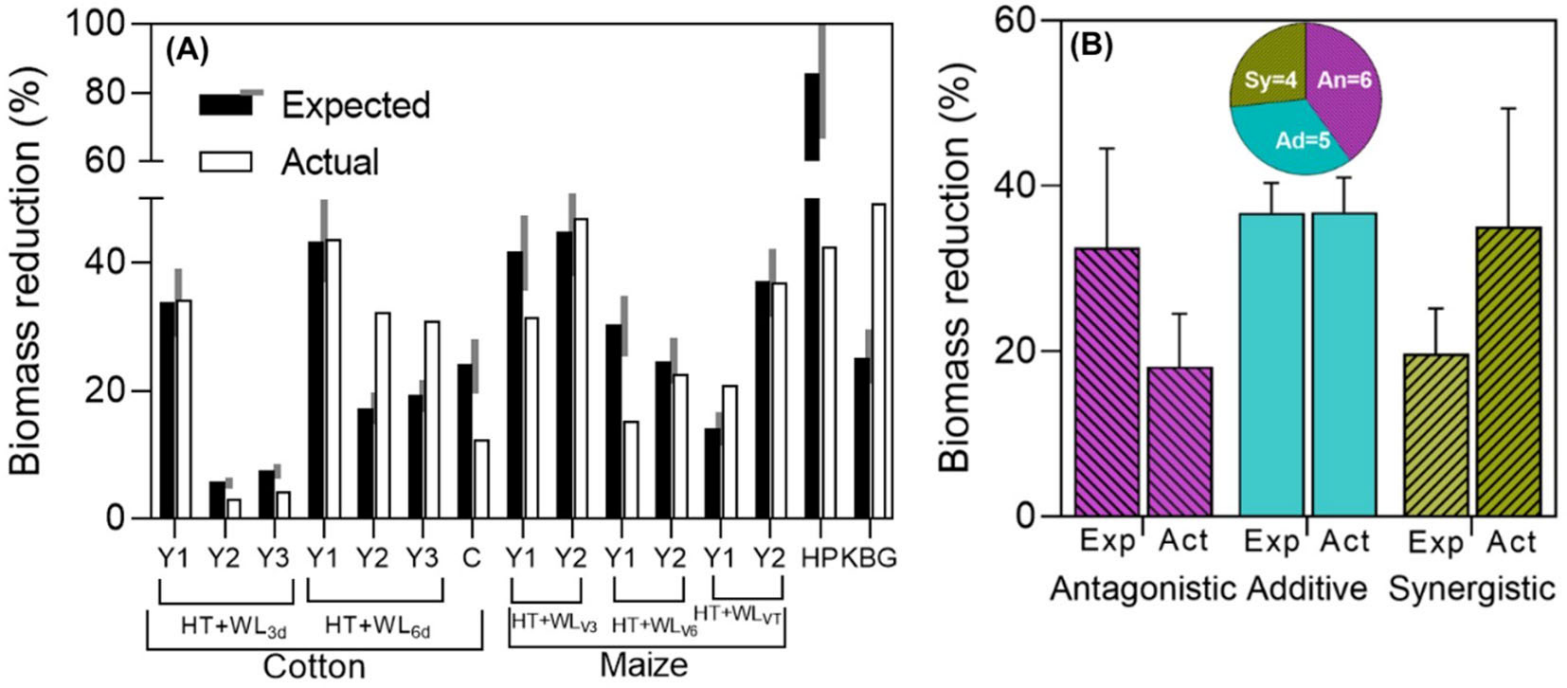
**(A)** Expected (assuming additive waterlogging and high temperature effects) and actual biomass reductions for the combined stresses (% of their respective controls) in cotton [one experiment that had 2 waterlogging durations, HT+WL_3d_ or HT+WL_6d_, in 3 experimental years (Y1, Y2, Y3; Wang et al., 2018, with intermittent stresses) and another study in cotton of 1 experimental year (C; Wang et al., 2024, with sequential stresses)], maize [3 timings of stress, HT+WL_V3_, HT+WL_V6_ or HT+WL_VT_, in 2 experimental years (Y1, Y2); Shao et al., 2023, with simultaneous stresses], hot pepper (HP; Lee et al., 2017, with intermittent stresses), Kentucky blue grass (KBG; Wang and Huang, 2004, with simultaneous stresses). Black and white bars represent expected and actual reductions within each of the experiments. The grey area on the side of the expected yield reduction represents a ±15% of its value and the effect was considered additive if the actual reduction was within this range, and antagonistic or synergistic if lower or higher than the shaded area. **(B)** Average (and standard error) reductions due to combined stress (relative to the controls) of the cases in which there were antagonistic, additive and synergistic effects (as seen in **A**). Expected (Exp) bars represent the expected effect on biomass of both stresses together assuming a strict additive effect (the sum of the reductions produced by waterlogging and by high temperatures acting separately), while actual (Act) bars represent the measured impact. Segments on top of the bars represent the standard error of the average effect of all observations corresponding to that particular type of interaction. Pie charts of studies with simultaneous, intermittent and sequential stresses are shown below, indicating the proportion of cases (and actual number of them, inside the pie portions) in which the effects were antagonistic (An), additive (Ad) and synergistic (Sy).

There were instances of antagonistic, additive, and synergistic interactions observed (see **Figure 2**), even within the same species. Indeed, in the two studies that reported effects across different seasons and types of treatments (timings, durations), all three types of interactions were observed for biomass in both cotton and maize, both extensive field crops (see **Figure 2A**). The single case reported for hot pepper and the study in cotton seedlings, both with intermittent stresses, showed that the actual penalty of the combined effects of waterlogging and high temperature was much lower than expected additively (see **Figure 2A**, HP and C), indicating a strongly antagonistic interaction in this instance. Conversely, the biomass of Kentucky bluegrass, an extensive pasture, was significantly more affected by the combined effects of waterlogging and high temperature than expected additively (see **Figure 2A**, KBG), representing a case of strong synergistic interaction. With the very few data available for shoot biomass, it seemed that the degree of synchrony in the imposition of the two stresses does not determine the type of interaction between them (**Supplementary Figure S2**).

Overall, when pooling the individually analyzed cases (**Figure 2A**) and considering the interactions of waterlogging and high temperatures on biomass and the type of stress combination (simultaneous, intermittent or sequential), there was a relatively similar distribution among the three types of interactions (**Figure 2B**; **Supplementary Figure S2**). This suggests that in some instances, synergistic effects on total growth might be expected, but may not necessarily translate into similar interactions observed for yield (compare **Figures 2A** and **1**). This discrepancy could be due, in part, to the possibility that the stresses considered may impact yield not only through effects on plant growth but also through direct effects on reproduction (see **Box 2**).

Box 2 **Differences between crops on stress effects on yield and biomass.**

**Figure B2:**
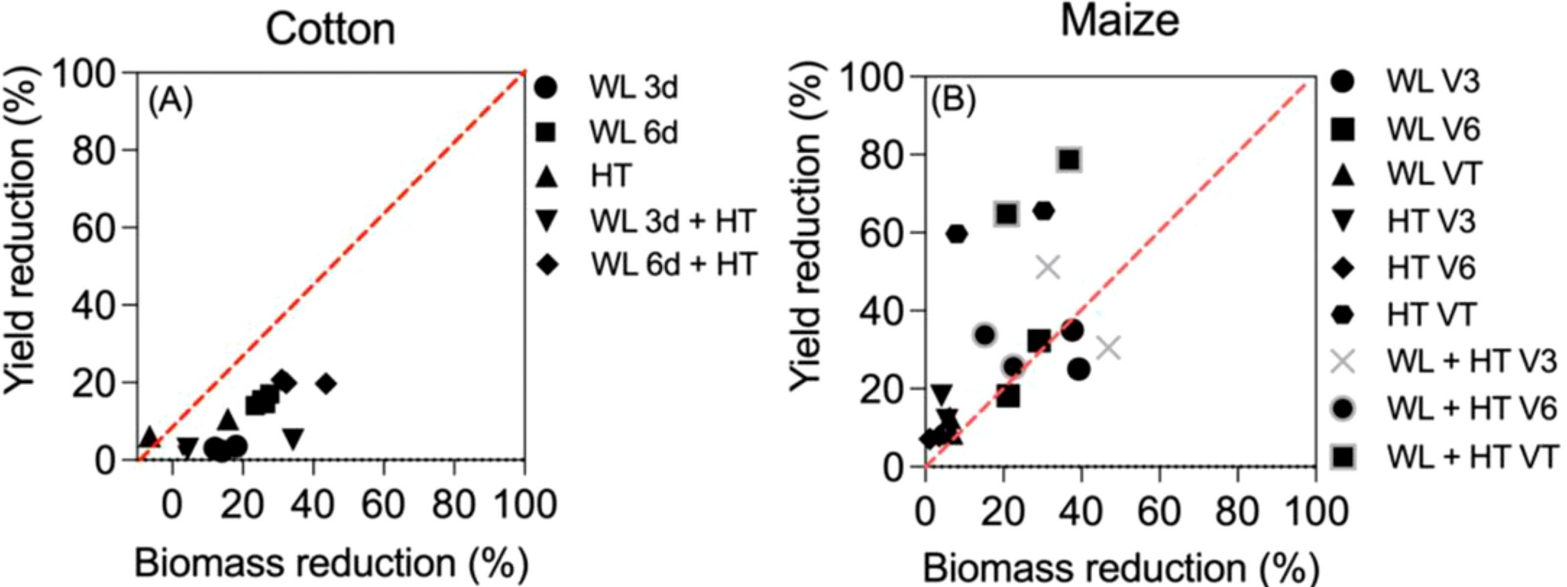
Yield vs biomass reductions (expressed as % of unstressed controls) in cotton **(A)** and maize **(B)**. In **(A)** cotton was subjected to waterlogging for 3 or 6 days (WL_3d_ and WL_6d_), high temperatures (HT) or combined stresses (WL_3d_+HT and WL_6d_+HT) during 3 experimental years (Wang et al., 2019). In **(B)** maize was subjected to waterlogging at V3, V6 or VT (WL_V3_, WL_V6_ and WL_VT_), high temperatures (HT_V3_, HT_V6_, HT_VT_) and combined stresses (WL+HT_V3_, WL+HT_V6_ and WL+HT_VT_) during 2 experimental years (Shao et al., 2023). The red dotted line indicates 1:1 relation.

**Figure 3:**
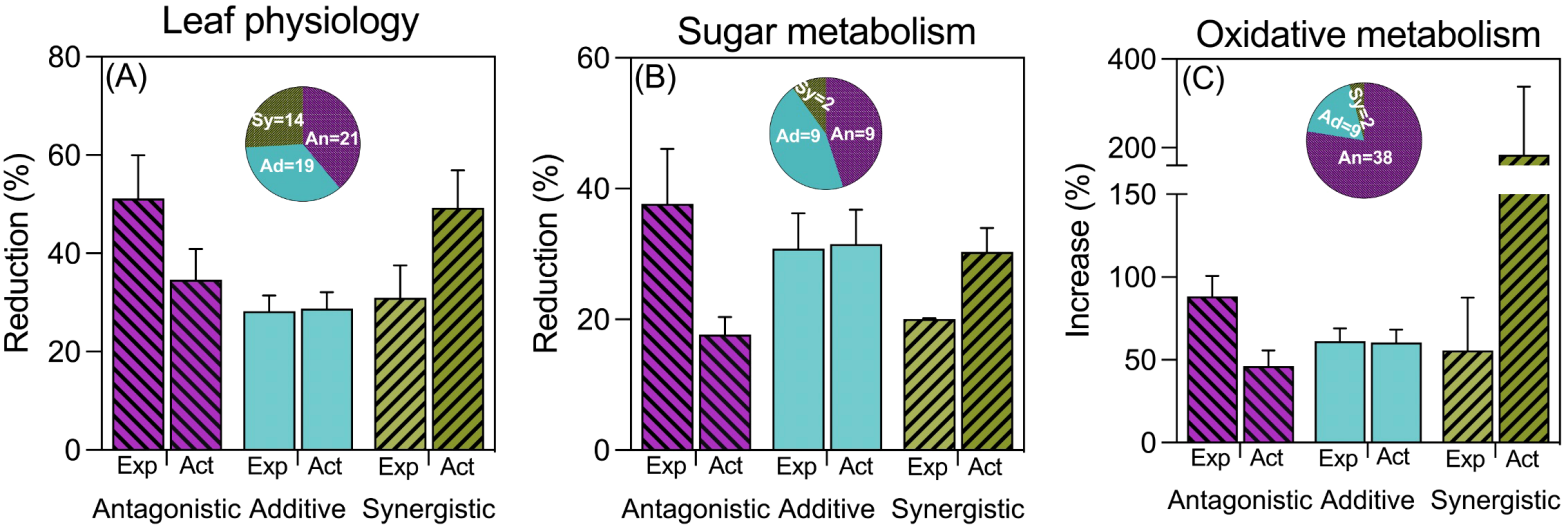
Reductions due to combined stress (expressed as % of controls) of antagonistic, additive and synergistic cases of leaf physiology **(A)**, sugar metabolism **(B)** and oxidative metabolism (ROS and detoxifying enzymes) **(C)**. Note that in the case of **(C)**, the stress effect is measured as an increase compared to controls. Expected (Exp) bars represent the expected effect of combined stress of the measured variables assuming a strict additive effect (the sum of the reductions produced by waterlogging and by high temperatures acting separately), while actual (Act) bars represent the actual (real) impact of combined stress Bars represent the average and standard error of *n* observations, which are described in the wheel chart inserted in each graph.

A more detailed analysis can be done in the two studies in which the effects of waterlogging, heat and both stresses together on yield and biomass were reported (**Figure B2**). Although in both crops there were some synergistic effects of the two stresses in biomass that were not evident in yield (*cf*. **Figures 2A** and **1**), there seemed to represent two distinct situations, even when in general -and expectedly- there was an overall positive trend between the reduction in yield and that in biomass (**Figure B2**). When considering the effects of all treatments (the effects of waterlogging and heat stress both in isolation and together), the effects in cotton were clearly stronger on biomass than on yield (with the only minor exception of two cases when heat stress slightly reduced yield when it had, bewilderingly though slightly, increased biomass; **Figure B2A** bottom left). On the other hand, in maize it was the opposite: in most cases the penalty on yield exceeded clearly that in biomass (**Figure B2B**). The difference might well be related to the many differences in background conditions in which the treatments were imposed, but would also reflect the dominance of the reproductive organs responsible of holding yield over other organs of the crops. While in cotton, as in most field crops, once reproduction is triggered, there is a sort of dominance of reproductive over vegetative organs (Pettigrew and Gerik, 2007; Rehman and Farooq, 2019), and therefore in the event of stresses affecting growth the reproductive organs may be less affected, in maize what it is harvested is an axillary organ (the apical dominance is exerted by the panicle that does only have male flowers and grain yield is concentrated in the female ear) and therefore more prone to be affected by stresses than vegetative organs (Andrade et al., 1999; Borrás and Vitantonio-Mazzini, 2018). In addition, maize reproduction has been shown to be extremely sensitive to stresses, particularly during the ‘critical period for grain number determination’ (around silking (Otegui et al., 2021) and several references therein), and yield may even collapse when the crop is exposed to severe stresses at that stage whilst biomass is much less affected (e.g., Rattalino et al., 2011; Ordóñez et al., 2015). Not surprisingly, the most conspicuous cases of departure from the 1-to-1 line in **Figure 3B** are those corresponding to the treatments imposed at tasseling, within the critical period of maize (indeed, data-points of treatments imposed at V3 and V6 seem much closer to the 1:1 line (**Figure B2B**).

There was a noticeable trend, in line with initial assumptions, where the actual shoot biomass penalties were larger in cases where the interaction was synergistic (averaging 35% with maximum penalties of 49%) compared to cases where it was antagonistic (averaging 32% with maximum penalties of 44%) (**Figure 2B**). Unexpectedly, we also found the reverse trend for expected additive effects: they were much larger in cases where actual effects were antagonistic than in cases where they were synergistic when pooling data from these response types. In essence, it became apparent that the larger the expected reductions due to combined stress, the smaller the actual combined effect.

## Leaf physiology and metabolism

Despite the primary objective of studying the effects of waterlogging, high temperatures, and other abiotic stresses on plants is related to improving yield resilience in agricultural crops (even when studying uncultivated model species), the majority of studies have focused on traits at the leaf and sub-leaf levels of organization. This focus is likely due to the assumption that (i) yield is primarily affected through these lower-level traits, and (ii) experiments at these levels are more manageable (including their feasibility in controlled conditions) and less resource-intensive. Therefore, it was unsurprising that when reviewing the literature to identify papers reporting on the effects of waterlogging and high temperatures both individually and in combination, the majority of studies concentrated on responses related to leaf physiological and metabolic traits (see **Table 1**).

When examining leaf physiological processes, the frequency and type of interaction between waterlogging and high temperatures varied depending on the specific trait considered (for details please see **Supplementary Table S1**). In most cases, the combined effect on chlorophyll content resembled the expected additive effect of each stress in isolation. Conversely, for leaf area, antagonistic interactions were most common, while synergistic interactions were predominant for leaf photosynthesis (see **Supplementary Table S1**). Across all traits categorized under ‘leaf physiology’, there were more instances of antagonistic interactions compared to synergistic ones, with additive effects falling in between (see **Figure 3A**).

Regarding sugar metabolism, soluble sugars exhibited diverse interactions, with a similar proportion of antagonistic, synergistic, and additive effects (see **Supplementary Table S2**). In contrast, leaf starch content predominantly showed antagonistic interactions, with a few additive cases and no synergistic effects (see **Supplementary Table S2**). For enzymes related to sucrose synthesis, most interactions were additive in the case of sucrose synthase, with a couple of antagonistic observations (see **Supplementary Table S2**). When pooling all traits within this category, we found a similar number of additive and antagonistic interactions, but very few cases of synergistic effects (see **Figure 3B**).

Compounds related to oxidative stress (i.e., MDA, ROS, and detoxifying enzymes) exhibited the most antagonistic interactions by far, indicating a significantly lower impact of combined stresses compared to the sum of each stress individually (**Figure 3C**). In more detail, the only trait that showed a similar number of additive and antagonistic interactions was the malondialdehyde (MDA) content in leaves, which also had a few cases of synergistic effects (**Supplementary Table S3**). Other traits, such as singlet oxygen and peroxide oxygen contents in leaf tissues, as well as the activities of detoxifying enzymes, predominantly showed antagonistic interactions between waterlogging and high temperature (**Supplementary Table S3**). If we analyze the data considering the synchronization between the imposition of the different stresses, the general picture is the same to that described for the complete dataset not considering whether the two stresses were imposed simultaneously, sequentially or intermittently (**Supplementary Figure S3**).

Although there was variation when considering particular traits, considering all traits pooled within the three groups belonging to leaf physiology and metabolism, the majority of cases (71 out of 127) reflected an antagonistic behavior, while only relatively few (18 out of 127) exhibited a synergistic one (**Figure 3**).

Similar to what was shown for biomass, when considering the three categories of leaf physiology and metabolism traits, there were contrasting trends for the expected and actual penalties: the actual reductions tended to increase (as presumed), and the expected reductions tended to decrease (beyond any presumed trend) from antagonistic to synergistic effects (**Figure 3**). This consequent negative relationship between the expected and actual magnitudes of reductions (or increases, in the case of oxidative stress traits) emphasizes that it is more likely that exposure to waterlogging would trigger mechanisms conferring some sort of tolerance to high temperature (or other stresses) if the penalties imposed by the individual stresses were severe.

## Hypothesis proposed

Reviewing the rather scarce literature considering the effects of waterlogging and high temperature both in isolation and combined shows that most of the interactions between these two stressors are antagonistic, with only a few synergistic ones. Analyzing separately species used in extensive and intensive agriculture did not change the overall trend, neither considering separately experiments in which the two stressors were imposed simultaneously, intermittently, or sequentially. There is no clear reason why exposure to anoxia would mitigate the effects of heat stress, which is reflected not only in intermittent stresses, but also when waterlogging and high temperatures are applied simultaneously. We, therefore, propose the hypothesis that waterlogging and high temperatures interact antagonistically; even though based on previous evidence about combined stresses like waterlogging and salinity, a synergistic interaction would have been expected (Barrett-Lennard, 2003; Yin et al., 2017). However, aligning with circumstantial evidence from the model plant *Arabidopsis thaliana*, it is suggested that previous exposure to anoxia might improve tolerance to high temperatures by promoting the expression of heat shock proteins (Banti et al., 2010). Moreover, most studies analyzing the effect of waterlogging and high temperatures occurring simultaneously have found fewer signs of stress acclimation than those subjecting plants to one stress followed by the other. To understand whether a prior stress can confer general tolerance to plants, enabling them to better withstand subsequent stress, experiments with successive stress events are necessary. These experiments should determine if plants can develop a generic capacity to tolerate different types of stress later on. For researchers focused on crop yields and food security, it is crucial that these experiments grow plants to maturity and measure the effects on yield of a crop in the field (or as close as possible to that condition). This emphasis on yield is critical because responses observed at lower levels of organization -or even in total biomass- may not necessarily correlate with crop yield (Passioura, 2006, 2020; Sadras and Richards, 2014). Sadras et al. (2020) emphasized that oversimplification and reductionism -both of which are inherent when measuring traits at the organ level of organization, especially if measurements are taken at stages not critical for yield determination and in isolated plants- can compromise the validity of conclusions relevant to crop yield.

## Conclusion

In this ‘Hypothesis article’, we highlighted a significant gap in understanding how waterlogging and high temperatures interact to influence crop yields. While the individual impacts of these stresses are well-studied, research on their combined effects remains extremely limited. Our review identified only 15 rigorous studies, most of which focused narrowly on leaf-level traits rather than crop yield -a critical oversight. Based on the empiric evidence from the limited available research, we hypothesize that waterlogging and high temperatures interact antagonistically, with one stress most often mitigating the adverse effects of the other. To close this knowledge gap and rigorously test this hypothesis, future research must prioritize crop yield as the primary outcome and focus on the most critical crops for global food security.

## Data Availability

The original contributions presented in the study are included in the article/**Supplementary Material**. Further inquiries can be directed to the corresponding author.
