# Supplementary material for: Dual stress, equivalent harm? hypothesizing on the type of interactions between waterlogging and high temperature
Source: Front Plant Sci. 2025 Jan 27;15:1472665. doi: 10.3389/fpls.2024.1472665 (PMC11807965; doi:10.3389/fpls.2024.1472665)
Supplement: Supplementary file 1 [file SupplementaryFile1.docx]

Supplementary materials

**Supplementary Table S1**. Reductions produced by the imposition of waterlogging (WL), high temperatures (HT) and both stresses combined (WL+HT) on different leaf physiological traits, categorized according to both (i) the type of crops (extensive field crops or intensive horticultural crops) and (ii) whether the two stresses were imposed simultaneously, sequentially or intermittently. In all cases the reductions are expressed as a percentage of the unstressed control [(control-stressed)/control)*100]. The cells corresponding to the combined effect are colored depending on whether the interaction between the two stresses were antagonistic (green) or synergistic (pink), the uncolored cells represent cases in which the effects of both stresses were additive.

| Reference | Trait | Type of crops | Simultaneous, sequential or intermittent stresses | WL | HT | WL+HT |
| --- | --- | --- | --- | --- | --- | --- |
|  |  |  |  | % | | |
| Liu et al. (2023) | Chlorophyll content (SPAD) | Intensive | Simultaneous | 45.9 | 51.3 | 78.4 |
| Lin et al. (2015a) |  | Intensive | Simultaneous | 27.5 | 12.3 | 45.3 |
| Lin et al. (2015a) |  | Intensive | Simultaneous | 5.3 | 51.1 | 58.9 |
| Lin et al. (2015a) |  | Intensive | Simultaneous | 39.2 | 48.8 | 70.6 |
| Lin et al. (2015b) |  | Intensive | Simultaneous | 3.9 | 7.9 | 13.2 |
| Lin et al. (2015b) |  | Intensive | Simultaneous | 1.3 | 10.4 | 15.6 |
| Wang et al. (2019) |  | Extensive | Intermittent | 10.1 | 6.1 | 15.5 |
| Wang et al. (2019) |  | Extensive | Intermittent | 23.0 | 6.1 | 29.0 |
| Shao et al. (2024) |  | Extensive | Simultaneous | 10.7 | 3.6 | 12.5 |
| Shao et al. (2024) |  | Extensive | Simultaneous | 10.7 | 3.6 | 12.5 |
| Shao et al. (2024) |  | Extensive | Simultaneous | 5.4 | 5.4 | 10.7 |
| Shao et al. (2024) |  | Extensive | Simultaneous | 16.7 | 5.0 | 20.0 |
| Shao et al. (2024) |  | Extensive | Simultaneous | 8.3 | 3.3 | 13.3 |
| Shao et al. (2024) |  | Extensive | Simultaneous | 3.3 | 3.3 | 8.3 |
| Wang et al. (2024) |  | Extensive | Sequential | 7.7 | 18.1 | 12.1 |
| Liu et al. (2023) | Fv/Fm | Intensive | Simultaneous | 18.9 | 31.1 | 39.2 |
| Lin et al. (2015a) |  | Intensive | Simultaneous | 62.4 | 13.2 | 99.8 |
| Lin et al. (2015a) |  | Intensive | Simultaneous | 13.7 | 70.6 | 100 |
| Lin et al. (2015a) |  | Intensive | Simultaneous | 72.5 | 99.9 | 100 |
| Lin et al. (2016) |  | Intensive | Simultaneous | 2.5 | 0.0 | 25.0 |
| Lin et al. (2016) |  | Intensive | Simultaneous | 21.3 | 31.3 | 68.7 |
| Lee et al. (2017) | Leaf area | Intensive | Intermittent | 36.9 | 45.7 | 46.4 |
| Wang et al. (2019) |  | Extensive | Intermittent | 1.9 | 12.5 | 11.1 |
| Wang et al. (2019) |  | Extensive | Intermittent | 1.9 | 17.3 | 5.4 |
| Wang et al. (2019) |  | Extensive | Intermittent | 5.4 | 14.3 | 4.2 |
| Wang et al. (2019) |  | Extensive | Intermittent | 29.8 | 12.5 | 44.5 |
| Wang et al. (2019) |  | Extensive | Intermittent | 34.5 | 17.3 | 43.6 |
| Wang et al. (2019) |  | Extensive | Intermittent | 36.2 | 14.3 | 38.0 |
| Wang et al. (2024) |  | Extensive | Sequential | 5.4 | 16.2 | 9.6 |
| Wang et al. (2018) | Leaf N content | Extensive | Intermittent | 8.7 | 8.7 | 0.0 |
| Wang et al. (2018) |  | Extensive | Intermittent | 30.4 | 8.7 | 43.48 |
| Wang et al. (2018) |  | Extensive | Intermittent | 25.0 | 8.3 | 37.50 |
| Liu et al. (2023) | Photosynthesis | Intensive | Simultaneous | 71.4 | 50.0 | 92.86 |
| Lee et al. (2017) |  | Intensive | Intermittent | 15.8 | 5.1 | 11.73 |
| Chen et al. (2017) |  | Extensive | Intermittent | 10.5 | 7.9 | 39.47 |
| Chen et al. (2017) |  | Extensive | Intermittent | 12.5 | 10.0 | 42.50 |
| Chen et al. (2017) |  | Extensive | Intermittent | 10.0 | 10.0 | 42.50 |
| Chen et al. (2017) |  | Extensive | Intermittent | 26.3 | 7.9 | 63.16 |
| Chen et al. (2017) |  | Extensive | Intermittent | 25.0 | 10.0 | 60.00 |
| Chen et al. (2017) |  | Extensive | Intermittent | 20.0 | 10.0 | 62.50 |
| Shao et al. (2024) |  | Extensive | Simultaneous | 24.3 | 10.8 | 27.0 |
| Shao et al. (2024) |  | Extensive | Simultaneous | 18.9 | 0.0 | 21.6 |
| Shao et al. (2024) |  | Extensive | Simultaneous | 25.0 | 20.3 | 37.5 |
| Shao et al. (2024) |  | Extensive | Simultaneous | 25.0 | 9.4 | 25.0 |
| Shao et al. (2024) |  | Extensive | Simultaneous | 6.3 | 25.0 | 43.8 |
| Wang et al. (2017) | Rubisco | Extensive | Intermittent | 16.7 | 14.6 | 18.75 |
| Wang et al. (2017) |  | Extensive | Intermittent | 6.3 | 22.9 | 14.58 |
| Wang et al. (2017) |  | Extensive | Intermittent | 22.9 | 14.6 | 37.50 |
| Wang et al. (2017) |  | Extensive | Intermittent | 22.5 | 17.5 | 25.00 |
| Wang et al. (2017) |  | Extensive | Intermittent | 31.3 | 22.9 | 39.58 |
| Shao et al. (2024) |  | Extensive | Simultaneous | 25.8 | 11.8 | 36.5 |
| Shao et al. (2024) |  | Extensive | Simultaneous | 21.9 | 6.2 | 27.5 |
| Shao et al. (2024) |  | Extensive | Simultaneous | 7.3 | 1.7 | 18.5 |
| Shao et al. (2024) |  | Extensive | Simultaneous | 20.4 | 8.3 | 23.1 |
| Shao et al. (2024) |  | Extensive | Simultaneous | 17.1 | 4.2 | 23.1 |
| Shao et al. (2024) |  | Extensive | Simultaneous | 8.8 | 14.4 | 19.0 |

**Supplementary Table S2**. Reductions produced by the imposition of waterlogging (WL), high temperature (HT) and both stresses combined (WL+HT) on different sugar metabolism traits, categorized according to both (i) the type of crops (extensive field crops or intensive horticultural crops) and (ii) whether the two stresses were imposed simultaneously, sequentially or intermittently. In all cases the reductions are expressed as a percentage of the unstressed control [(control-stressed)/control)*100]. The cells corresponding to the combined effect are colored depending on whether the interaction between the two stresses were antagonistic (green) or synergistic (pink), the uncolored cells represent cases in which the effects of both stresses were additive.

| Reference | Trait | Type of crops | Simultaneous, sequential or intermittent stresses | WL | HT | WL+HT |
| --- | --- | --- | --- | --- | --- | --- |
| Liu et al. (2023) | Soluble sugar leaf content | Intensive | Simultaneous | 6.4 | 13.8 | 34.0 |
| Liu et al. (2023) |  | Intensive | Simultaneous | 6.6 | 9.5 | 16.4 |
| Wang et al. (2017) |  | Extensive | Intermittent | 10.0 | 3.3 | 10.0 |
| Wang et al. (2017) |  | Extensive | Intermittent | 10.3 | 10.3 | 13.8 |
| Wang et al. (2017) | Starch leaf content | Extensive | Intermittent | 8.3 | 11.1 | 22.2 |
| Wang et al. (2017) |  | Extensive | Intermittent | 9.4 | 3.1 | 9.4 |
| Wang et al. (2017) |  | Extensive | Intermittent | 9.7 | 9.7 | 22.6 |
| Wang et al. (2017) |  | Extensive | Intermittent | 27.8 | 11.1 | 27.8 |
| Wang et al. (2017) |  | Extensive | Intermittent | 25.0 | 3.1 | 22.9 |
| Wang et al. (2017) |  | Extensive | Intermittent | 29.0 | 9.7 | 29.0 |
| Chen et al. (2017) | Sucrose synthase | Extensive | Intermittent | 16.7 | 33.3 | 50.0 |
| Chen et al. (2017) |  | Extensive | Intermittent | 33.3 | 55.6 | 11.1 |
| Chen et al. (2017) |  | Extensive | Intermittent | 0.0 | 40.0 | 40.0 |
| Chen et al. (2017) |  | Extensive | Intermittent | 16.7 | 33.3 | 50.0 |
| Chen et al. (2017) |  | Extensive | Intermittent | 11.1 | 55.6 | 11.1 |
| Chen et al. (2017) |  | Extensive | Intermittent | 10.0 | 40.0 | 50.0 |
| Chen et al. (2017) | Sucrose phosphate synthase | Extensive | Intermittent | 6.3 | 6.3 | 12.5 |
| Chen et al. (2017) |  | Extensive | Intermittent | 20.0 | 0.0 | 20.0 |
| Chen et al. (2017) |  | Extensive | Intermittent | 25.0 | 6.3 | 25.0 |
| Chen et al. (2017) |  | Extensive | Intermittent | 20.0 | 0.0 | 26.7 |

**Supplementary Table S3**. Increases produced by the imposition of waterlogging (WL), high temperature (HT) and both stresses combined (WL+HT) on different traits relative to oxidative stress (i.e. MDA, ROS and detoxifying enzymes), categorized according to both (i) the type of crops (extensive field crops or intensive horticultural crops) and (ii) whether the two stresses were imposed simultaneously, sequentially or intermittently. In all cases the increases are expressed as a percentage of the unstressed control [(control-stressed)/control)*100]. The cells corresponding to the combined effect are colored depending on whether the interaction between the two stresses were antagonistic (green) or synergistic (pink), the uncolored cells represent cases in which the effects of both stresses were additive.

| Reference | Trait | Type of crops | Simultaneous, sequential or intermittent stresses | WL | HT | WL+HT |
| --- | --- | --- | --- | --- | --- | --- |
| Liu et al. (2023) | MDA | Intensive | Simultaneous | 41.4 | 44.8 | 89.7 |
| Xu et al. (2021) |  | Extensive | Intermittent | 0.0 | 157.1 | 357.1 |
| Wang et al. (2019) |  | Extensive | Intermittent | 12.0 | 60.0 | 44.0 |
| Wang et al. (2019) |  | Extensive | Intermittent | 30.4 | 100.0 | 82.6 |
| Wang et al. (2019) |  | Extensive | Intermittent | 20.0 | 85.0 | 55.0 |
| Wang et al. (2019) |  | Extensive | Intermittent | 28.0 | 60.0 | 16.0 |
| Wang et al. (2019) |  | Extensive | Intermittent | 65.2 | 100.0 | 47.8 |
| Wang et al. (2019) |  | Extensive | Intermittent | 50.0 | 85.0 | 35.0 |
| Shao et al. (2024) |  | Extensive | Simultaneous | 72.8 | 11.3 | 79.2 |
| Shao et al. (2024) |  | Extensive | Simultaneous | 60.4 | 15.2 | 68.9 |
| Shao et al. (2024) |  | Extensive | Simultaneous | 13.4 | 25.8 | 37.5 |
| Shao et al. (2024) |  | Extensive | Simultaneous | 57.5 | 4.3 | 63.1 |
| Shao et al. (2024) |  | Extensive | Simultaneous | 47.4 | 3.1 | 50.5 |
| Shao et al. (2024) |  | Extensive | Simultaneous | 5.2 | 18.8 | 30.2 |
| Wang et al. (2024) |  | Extensive | Sequential | 12.0 | 60.0 | 36.0 |
| Liu et al. (2023) | ^1^O_2_ | Intensive | Simultaneous | 151.9 | 103.7 | 196.3 |
| Wang et al. (2019) |  | Extensive | Intermittent | 9.1 | 45.5 | 15.2 |
| Wang et al. (2019) |  | Extensive | Intermittent | 36.4 | 90.9 | 45.5 |
| Wang et al. (2019) |  | Extensive | Intermittent | 0.0 | 23.3 | 0.0 |
| Wang et al. (2019) |  | Extensive | Intermittent | 36.4 | 45.5 | 42.4 |
| Wang et al. (2019) |  | Extensive | Intermittent | 81.8 | 90.9 | 131.8 |
| Wang et al. (2019) |  | Extensive | Intermittent | 33.3 | 23.3 | 40.0 |
| Wang et al. (2024) |  | Extensive | Sequential | 6.1 | 51.5 | 39.4 |
| Liu et al. (2023) | H_2_O_2_ | Intensive | Simultaneous | 81.8 | 263.6 | 272.7 |
| Lin et al. (2015b) |  | Intensive | Simultaneous | 37.5 | 50.0 | 337.5 |
| Wang et al. (2019) |  | Extensive | Intermittent | 6.7 | 20.0 | 6.7 |
| Wang et al. (2019) |  | Extensive | Intermittent | 4.3 | 30.4 | 17.4 |
| Wang et al. (2019) |  | Extensive | Intermittent | 18.5 | 25.9 | 29.6 |
| Wang et al. (2019) |  | Extensive | Intermittent | 20.0 | 20.0 | 33.3 |
| Wang et al. (2019) |  | Extensive | Intermittent | 17.4 | 30.4 | 30.4 |
| Wang et al. (2024) |  | Extensive | Sequential | 0.0 | 45.8 | 33.3 |
| Wang et al. (2019) | SOD | Extensive | Intermittent | 18.0 | 19.0 | 4.0 |
| Wang et al. (2019) |  | Extensive | Intermittent | 3.4 | 34.5 | 12.1 |
| Wang et al. (2019) |  | Extensive | Intermittent | 10.0 | 20.0 | 0.0 |
| Wang et al. (2019) |  | Extensive | Intermittent | 10.0 | 19.0 | 22.0 |
| Wang et al. (2019) |  | Extensive | Intermittent | 22.4 | 34.5 | 20.7 |
| Wang et al. (2019) |  | Extensive | Intermittent | 10.0 | 20.0 | 12.0 |
| Wang et al. (2024) |  | Extensive | Sequential | 18.0 | 10.0 | 28.0 |
| Wang et al. (2019) | CAT | Extensive | Intermittent | 31.3 | 56.3 | 25.0 |
| Wang et al. (2019) |  | Extensive | Intermittent | 20.0 | 140.0 | 40.0 |
| Wang et al. (2019) |  | Extensive | Intermittent | 31.8 | 18.2 | 40.9 |
| Wang et al. (2019) |  | Extensive | Intermittent | 56.3 | 56.3 | 87.5 |
| Wang et al. (2019) |  | Extensive | Intermittent | 80.0 | 140.0 | 110.0 |
| Wang et al. (2019) | POX | Extensive | Intermittent | 3.4 | 28.8 | 3.4 |
| Wang et al. (2019) |  | Extensive | Intermittent | 36.0 | 32.0 | 24.0 |
| Wang et al. (2019) |  | Extensive | Intermittent | 0.0 | 16.7 | 1.9 |
| Wang et al. (2019) |  | Extensive | Intermittent | 10.2 | 28.8 | 18.6 |
| Wang et al. (2019) |  | Extensive | Intermittent | 44.0 | 32.0 | 52.0 |
| Wang et al. (2019) |  | Extensive | Intermittent | 14.8 | 16.7 | 29.6 |
| Wang et al. (2024) |  | Extensive | Sequential | 14.3 | 17.1 | 32.1 |

**Supplementary Figure S1.** Number of papers found through a survey conducted in the *Web of Science* database on February 28^th^, 2024 under ‘plant sciences’, ‘agronomy’ and ‘horticulture’ categories whose title included: i) ´high temperature´ or ´elevated temperature´ or ´heat´ (HT), ii) ´waterlogging´ or ´flooding´ (WL), iii) the combination of them: ´waterlogging and high temperature’, ‘waterlogging and elevated temperature’, ‘waterlogging and heat’, ‘flooding and high temperature’, ‘flooding and elevated temperature’, or ‘flooding and heat’ (WL+HT).


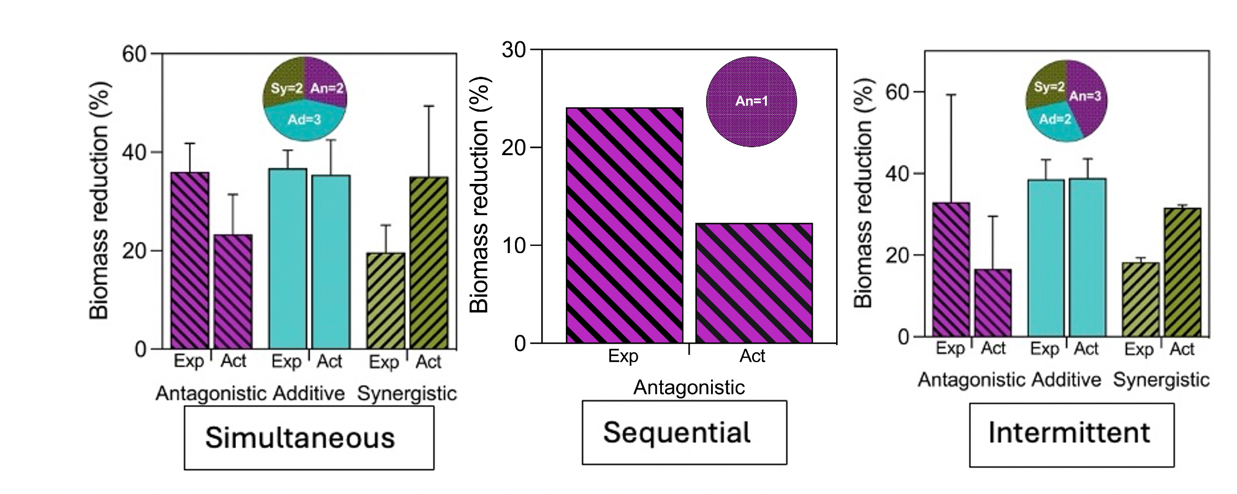


Figure S2. The three panels stand for data from experiments imposing both stresses either simultaneously, sequentially and intermittently. Expected (Exp) bars represent the expected effect on the traits considered of both stresses together assuming a strict additive effect (the sum of the reductions produced by waterlogging and by high temperatures acting separately), while actual (Act) bars represent the actual (real) impact of combined stress. Segments on top of the bars represent the standard error of the average effect of all observations corresponding to that particular type of interaction (figure inserted within the pie chart).


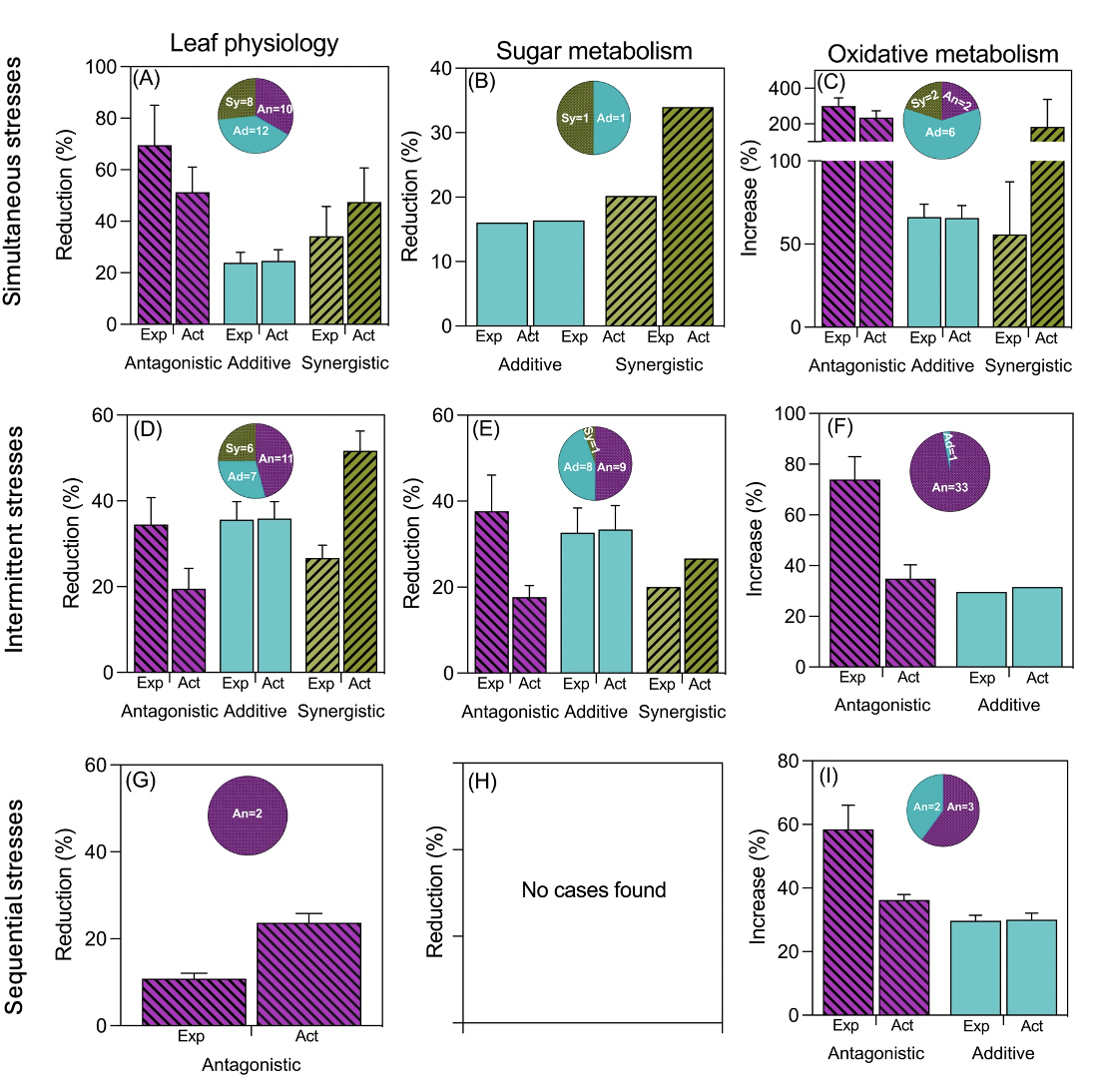


Figure S3. Reductions due to combined stress (expressed as % of controls) of antagonistic, additive and synergistic cases of leaf physiology (A, D, G), sugar metabolism (B, E, H) and oxidative metabolism (ROS and detoxifying enzymes) (C, F, I). The three rows of panels stand for data from experiments imposing both stresses either simultaneously (A-C), intermittently (D-F), and sequentially (G-H). Note that in the case of oxidative metabolism the stress effect is measured as an increase compared to controls. Also, in H, there were no data of sugar metabolism determined in the study applying sequential stresses; and in three other cases the interactions reported were antagonistic only (G) or antagonistic and additive (F, I). Expected (Exp) bars represent the expected effect on the traits considered of both stresses together assuming a strict additive effect (the sum of the reductions produced by waterlogging and by high temperatures acting separately), while actual (Act) bars represent the actual (real) impact of combined stress. Segments on top of the bars represent the standard error of the average effect of all observations corresponding to that particular type of interaction (figure inserted within the pie chart).
